# PPE Surface Proteins Are Required for Heme Utilization by *Mycobacterium tuberculosis*

**DOI:** 10.1128/mBio.01720-16

**Published:** 2017-01-24

**Authors:** Avishek Mitra, Alexander Speer, Kan Lin, Sabine Ehrt, Michael Niederweis

**Affiliations:** a Department of Microbiology, University of Alabama at Birmingham, Birmingham, Alabama, USA; b Department of Microbiology and Immunology, Weill Cornell Medical College, New York, New York, USA; Washington University School of Medicine

## Abstract

Iron is essential for replication of *Mycobacterium tuberculosis*, but iron is efficiently sequestered in the human host during infection. Heme constitutes the largest iron reservoir in the human body and is utilized by many bacterial pathogens as an iron source. While heme acquisition is well studied in other bacterial pathogens, little is known in *M. tuberculosis*. To identify proteins involved in heme utilization by *M. tuberculosis*, a transposon mutant library was screened for resistance to the toxic heme analog gallium(III)-porphyrin (Ga-PIX). Inactivation of the *ppe36*, *ppe62*, and *rv0265c* genes resulted in resistance to Ga-PIX. Growth experiments using isogenic *M. tuberculosis* deletion mutants showed that PPE36 is essential for heme utilization by *M. tuberculosis*, while the functions of PPE62 and Rv0265c are partially redundant. None of the genes restored growth of the heterologous *M. tuberculosis* mutants, indicating that the proteins encoded by the genes have separate functions. PPE36, PPE62, and Rv0265c bind heme as shown by surface plasmon resonance spectroscopy and are associated with membranes. Both PPE36 and PPE62 proteins are cell surface accessible, while the Rv0265c protein is probably located in the periplasm. PPE36 and PPE62 are, to our knowledge, the first proline-proline-glutamate (PPE) proteins of *M. tuberculosis* that bind small molecules and are involved in nutrient acquisition. The absence of a virulence defect of the *ppe36* deletion mutant indicates that the different iron acquisition pathways of *M. tuberculosis* may substitute for each other during growth and persistence in mice. The emerging model of heme utilization by *M. tuberculosis* as derived from this study is substantially different from those of other bacteria.

## IMPORTANCE

Tuberculosis is caused by *Mycobacterium tuberculosis* and is a devastating disease affecting eight million people each year. Iron is an essential nutrient for replication of *M. tuberculosis* in the human host. More than 70% of iron in the human body is bound in heme. Not surprisingly, many bacterial pathogens, including *M. tuberculosis*, are able to acquire iron from heme. However, the mechanism of heme uptake by *M. tuberculosis* is poorly understood. We have identified two novel surface proteins that bind heme and are required for heme utilization by *M. tuberculosis*. These findings constitute a major advancement of our understanding of iron acquisition by *M. tuberculosis* and show that *M. tuberculosis* has evolved heme uptake systems different from the paradigms established by other bacteria.

## INTRODUCTION

Tuberculosis is the leading cause of death from a single infectious disease, resulting in 1.5 million deaths in 2014 (1). *Mycobacterium tuberculosis* infects humans by aerosols and proliferates in alveolar macrophages by inhibiting macrophage maturation (2). Acquisition of iron within the human host is critical for replication and virulence of pathogenic bacteria (3), because iron is one of the least accessible micronutrients due to sequestration by host proteins (4). To obtain iron, *M. tuberculosis* produces small molecules with high affinity for iron, the mycobactins and carboxymycobactins (5). Secretion of siderophores by *M. tuberculosis* is dependent on the MmpL4/MmpS4 and MmpL5/MmpS5 membrane complexes (6, 7). While the initial step of siderophore uptake across the outer membrane is unknown (8), the IrtA/IrtB protein complex is required for efficient uptake of ferric carboxymycobactins across the inner membrane (9). Iron is reductively released from carboxymycobactins by the flavin adenine dinucleotide (FAD)-binding domain of IrtA (10), and the uncomplexed carboxymycobactins are subsequently recycled by secretion through the MmpS4/MmpL4 and MmpS5/MmpL5 transporters (7). However, it is estimated that approximately 70% of iron in the human body is tightly bound in heme (11), most often in a complex with hemoglobin (12), and cannot be solubilized by bacterial siderophores. Not surprisingly, many bacterial pathogens have developed mechanisms for iron acquisition from heme (13). In Gram-negative bacteria, host hemoproteins or heme are captured by outer membrane receptors that translocate heme into the periplasm in a TonB-dependent manner (14). Then, heme is transported across the inner membrane by specific permeases (11). Recent studies have shown that *M. tuberculosis* is also capable of utilizing heme as an iron source (15, 16). Rv0203 was identified as a heme-binding protein of *M. tuberculosis* with a limited role in heme utilization by *M. tuberculosis* (16). Rv0203 was shown to transfer heme to the extracellular domains of the inner membrane proteins MmpL3 and MmpL11. However, MmpL3 and MmpL11 are resistance-nodulation-cell division (RND)-type efflux pumps involved in export of trehalose monomycolate, lipids, or other lipid-like molecules for maintenance of the mycobacterial cell wall (17–19). Thus, the actual roles of MmpL3 and MmpL11 in heme utilization by *M. tuberculosis* are unclear. MhuD is an oxygen-dependent heme-degrading enzyme of *M. tuberculosis* involved in iron release from heme (20). Overall, the mechanisms by which *M. tuberculosis* imports heme into the cell are poorly understood. The main objective of this study was to identify proteins required for heme uptake in *M. tuberculosis*. Here, we demonstrate that PPE36, PPE62, and Rv0265c are novel heme-binding membrane proteins of *M. tuberculosis* that have nonredundant roles in heme utilization. We show that PPE36 and PPE62 are cell surface receptors for heme in *M. tuberculosis* and that PPE36 is essential for heme utilization *in vitro*.

## RESULTS

### Synthesis and characterization of gallium(III)-porphyrin.

**FIG 1:**
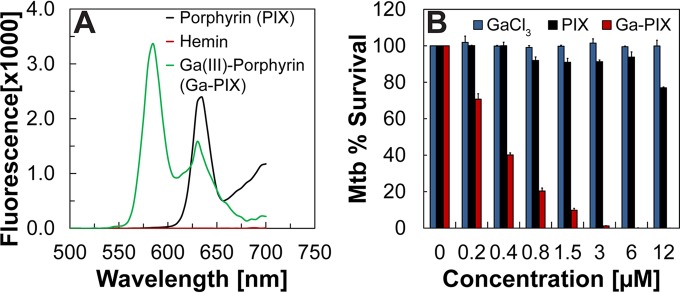
Characterization of gallium(III)-porphyrin (Ga-PIX) and its toxicity for *M. tuberculosis*. (A) Fluorescence spectra of PIX, hemin, and synthesized Ga-PIX after excitation at 410 nm. (B) Survival of *M. tuberculosis* (Mtb) in iron-free Middlebrook 7H9 medium containing 2 µM ferric carboxymycobactin (cMBT) and in the presence of increasing concentrations of gallium chloride (GaCl_3_), porphyrin IX (PIX), and gallium porphyrin IX (Ga-PIX) was determined by the microplate alamarBlue assay. Error bars represent standard errors of mean values of biological triplicates.

To identify unknown components required for heme uptake by *M. tuberculosis*, we exploited the toxicity of gallium(III) for bacteria. The structural properties and coordination chemistry of Ga(III) are similar to those of iron(III), and Ga(III) can substitute for Fe(III) as a cofactor in many proteins. However, Ga(III) cannot be reduced under physiological conditions and, therefore, cannot catalyze redox reactions (21). The efficacy of Ga(III) as an antimicrobial agent has been demonstrated in several bacterial pathogens (22), including *M. tuberculosis* (23). Since iron utilization from heme is achieved by removal of iron from the protoporphyrin (PIX) ring, we hypothesized that *M. tuberculosis* may similarly remove gallium from gallium(III)-porphyrin (Ga-PIX). The subsequent incorporation of Ga(III) into essential proteins would then be toxic for *M. tuberculosis*. Therefore, *M. tuberculosis* mutants resistant to Ga-PIX should have disruptions in essential components of the heme uptake and/or utilization pathways. To identify novel components of heme utilization by *M. tuberculosis*, we synthesized Ga-PIX from protoporphyrin IX (PIX) and gallium chloride as previously described (24) and obtained 30 mg of Ga-PIX. PIX, Fe(III)-PIX (hemin), and Ga(III)-PIX absorb light with a characteristic maximum at 410 nm but have distinct fluorescence emission spectra (25). While hemin does not exhibit any fluorescence, PIX and Ga(III)-PIX showed the characteristic fluorescence emission maxima at 585 nm and 610 nm, respectively (Fig. 1A). The synthesis of Ga(III)-PIX was further confirmed by distinct retention factors and fluorescence on silica gel thin-layer chromatography plates (see Fig. S1 in the supplemental material).

10.1128/mBio.01720-16.1 FIG S1  Characterization of gallium(III)-porphyrin (Ga-PIX) and Ga-PIX-resistant M. tuberculosis mutants. (A) Absorbance spectra of porphyrin (PIX) (black), hemin (red), and synthesized gallium(III)-porphyrin (Ga-PIX) (green). (B) Fluorescence determined by thin-layer chromatography. Protoporphyrin IX (PIX) (lane 1), hemin (lane 2), and gallium(III)-porphyrin (Ga-PIX) (lane 3) were separated on silica gel plates using a methanol-water mixture (3:1 volume ratio). Fluorescence was visualized using UV light (260 nm). (C) Growth of M. tuberculosis transposon mutants in HdB minimal medium containing either 2 µM ferric carboxymycobactin (cMBT) (black bars) or 10 µM hemin (red bars) as determined using a microplate alamarBlue assay (MABA). Lowercase letters b for Tn mutants indicate significantly different values compared to the value for the wild type (wt) (a) as determined by Tukey’s honestly significant difference (HSD) test following an *F* test (*P* < 0.009). (D) Growth of M. tuberculosis transposon mutants in HdB minimal medium containing either 2 µM cMBT (black bars) or 2 µM cMBT and 3 µM Ga-PIX (red bars) as determined using MABA. Error bars represent standard errors of mean values of biological triplicates. (E) DNA sequencing identified the transposon insertion sites in the genome of *M. tuberculosis* in the *M. tuberculosis* transposon mutants Tn*1*, Tn*2*, and Tn*5* resistant to Ga-PIX. The locations of genes in the *M. tuberculosis* H37Rv genome are denoted by blue arrows and numbers. Transposon insertion sites are denoted by red arrows and numbers. Download FIG S1, JPG file, 1.2 MB. Copyright © 2017 Mitra et al. 2017 Mitra et al. This content is distributed under the terms of the Creative Commons Attribution 4.0 International license.

Ga(III)-PIX was highly toxic toward *M. tuberculosis* with an MIC_90_ of ~1.5 µM in iron-free Middlebrook 7H9 medium supplemented with 2 µM ferric carboxymycobactin (cMBT) as the sole iron source (Fig. 1B). Ga(III) salts are known to be toxic for *M. tuberculosis* (23) and might be present in traces in our Ga-PIX preparation. However, *M. tuberculosis* was completely resistant to GaCl_3_ up to concentrations of 12 µM in our experiments (Fig. 1B), probably because the presence of cMBT preloaded with Fe(III) prevented Ga(III) from entering the cell, at least initially, and thereby reduced the toxicity of GaCl_3_. *M. tuberculosis* was also highly resistant to PIX (Fig. 1B). These results validated the synthesis of Ga(III)-PIX and demonstrate that Ga(III)-PIX is toxic for *M. tuberculosis*.

### Identification of *M. tuberculosis* genes involved in heme utilization.

To identify genes involved in heme utilization by *M. tuberculosis*, we constructed a transposon mutant library in the avirulent *M. tuberculosis* strain mc^2^6206 containing approximately 70,000 mutants. The *M. tuberculosis* transposon library was cultured on iron-free Middlebrook 7H10 agar supplemented with 3 µM Ga-PIX (~3× MIC_90_) and 2 µM ferric cMBT as the non-heme iron source. After incubation of the plates at 37°C for ~40 days, 10 clones were obtained; these clones were more resistant to Ga-PIX than wild-type (wt) *M. tuberculosis* as determined by a microplate alamarBlue assay (MABA) (Fig. S1D). The ability of these *M. tuberculosis* mutants to utilize heme was then examined in growth experiments. The *M. tuberculosis* Tn mutants grew like the parent *M. tuberculosis* strain in the presence of ferric cMBT as the sole iron source in liquid medium (Fig. S1C). The *M. tuberculosis* Tn*1*, Tn*2*, and Tn*5* mutants exhibited small, but significant, defects in heme utilization compared to the parent strain (Fig. S1C). Sequencing of the chromosomal DNA of the *M. tuberculosis* transposon mutants Tn*1* and Tn*2* revealed insertions in the intergenic regions of *rv0265c-rv0266c* genes and *pe22-ppe36* (*rv2107-rv2108*), respectively, suggesting that *rv0265c* and *ppe36* expression may be downregulated. The insertion in the Tn*5* mutant mapped at the start codon of *ppe62* (*rv3533c* gene) (Fig. S1E).

**FIG 2:**
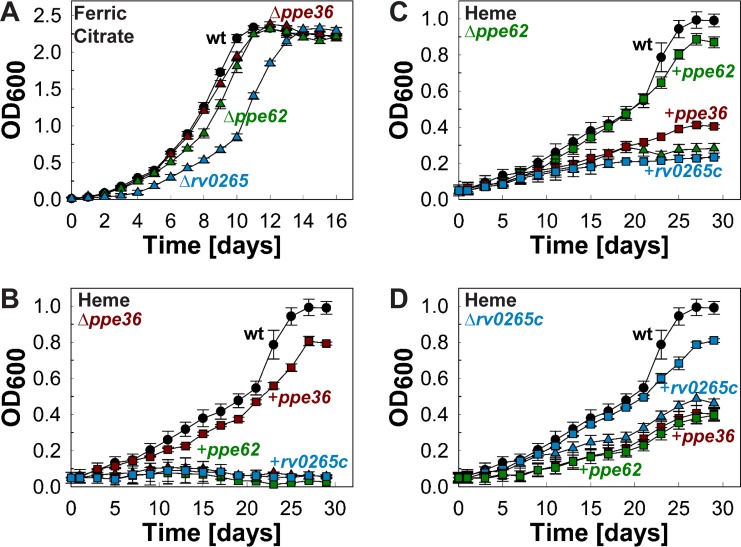
Identification of genes involved in heme utilization in *M. tuberculosis*. (A) Growth of wild-type (wt) *M. tuberculosis* mc^2^6206 (black circles), ML2411 (Δ*ppe36*) (red triangles), and ML2412 (Δ*ppe62*) (green triangles), and ML2413 (*rv0265c* deletion mutant) (blue triangles) in HdB minimal medium with 10 µM ammonium ferric citrate as the sole iron source. (B to D) Growth of wt *M. tuberculosis* mc^2^6206 (black circles), mutant (triangles), and complemented mutant (squares) in HdB minimal medium with 10 µM hemin as the sole iron source and 20 µM 2,2′-dipyridyl to prevent utilization of trace iron. Complementation of mutant with *ppe36*, *ppe62*, or *rv0265c* is shown in red, green, and blue squares, respectively. Error bars represent standard errors of mean values of biological triplicates.

To examine the functions of *rv0265c*, *ppe62*, and *ppe36*, isogenic deletion mutants were constructed by allelic exchange in the avirulent *M. tuberculosis* strain mc^2^6206 using a *gfp-hyg* cassette as schematically shown in Fig. S2. Due to the short intergenic region (80 bp) between the *rv0264c* and *rv0265c* genes, a promoter was inserted upstream of the *rv0264c* gene to prevent any polar effect from deletion of the *rv0265c* gene. After homologous recombination the *gfp-hyg* cassette was then excised by Cre recombinase, leaving a *loxP* site in lieu of the gene of interest. This process generated the unmarked *M. tuberculosis* mutants ML2411 (Δ*ppe36*::*loxP*), ML2412 (Δ*ppe62*::*loxP*), and ML2413 (Δ*rv0265c*::*loxP*) (Table S3). All mutant strains were verified by PCR (Fig. S2). The role of these genes in heme utilization by *M. tuberculosis* was analyzed by monitoring the growth of the *M. tuberculosis* mutants in HdB minimal medium (see Materials and Methods) with 10 µM ammonium ferric citrate or 10 µM hemin as the sole iron source. The medium containing hemin was supplemented with 20 µM 2,2′-dipyridyl (DIP) to prevent utilization of trace iron as described earlier (6). In separate experiments, we demonstrated that 20 µM DIP in medium with no iron added completely inhibits growth of *M. tuberculosis* (Fig. S3). Deletion of *ppe36* (ML2411) and *ppe62* (ML2412) did not affect growth of *M. tuberculosis* in medium with ammonium ferric citrate as the sole iron source, while the *rv0265c* deletion mutant had a slightly reduced growth rate compared to wt *M. tuberculosis* (Fig. 2A). In contrast, the Δ*ppe36* mutant did not grow in minimal medium with hemin (Fig. 2B), while growth of the *ppe62* and *rv0265c* deletion mutants was strongly reduced (Fig. 2C and D). The ability of all *M. tuberculosis* mutants to utilize hemin as an iron source was restored to wt levels when complemented with the respective gene (Fig. 2B to D).

10.1128/mBio.01720-16.2 FIG S2  Construction and validation of isogenic M. tuberculosis mutants. (A) Schematic representation of construction of *M. tuberculosis* mutants. Genomic DNA was extracted for clones after unmarking by Cre recombinase. Deletion of *ppe36* (B), *ppe62* (C), and Rv0265 (D) was confirmed by PCR using primers that bind outside the target open reading frame as indicated in panel A. All validation primers are listed in Table S2. Download FIG S2, JPG file, 1.1 MB. Copyright © 2017 Mitra et al. 2017 Mitra et al. This content is distributed under the terms of the Creative Commons Attribution 4.0 International license.

10.1128/mBio.01720-16.3 FIG S3  M. tuberculosis does not grow in iron-depleted medium containing an iron chelator. Growth of wt *M. tuberculosis* mc^2^6206 in iron-depleted medium supplemented with 10 µM ammonium ferric citrate or 10 µM hemin or with no iron shown in black, red, and blue circles, respectively. Cultures with heme or no iron include a 20 µM concentration of the iron chelator 2,2′-dipyridyl (DIP) to remove any residual iron in the medium. Error bars represent standard errors of mean values of biological triplicates. Download FIG S3, TIF file, 1.3 MB. Copyright © 2017 Mitra et al. 2017 Mitra et al. This content is distributed under the terms of the Creative Commons Attribution 4.0 International license.

Since the molecular functions of PPE36, PPE62, and Rv0265c are unknown and the Δ*ppe62* or Δ*rv0265c* mutants did not reveal a complete phenotype, we expressed all genes in the respective *M. tuberculosis* mutants and examined whether these genes could functionally substitute for each other. While providing a copy of the missing gene fully restored growth of each of the three *M. tuberculosis* mutants on heme, none of the other genes recovered growth (Fig. 2B to D). These results indicated that the PPE36, PPE62, and Rv0265c proteins have distinct functions in heme utilization by *M. tuberculosis*. This finding is consistent with the strong phenotypes of the single mutants with heme as the sole iron source. Increasing the hemin concentration to up to 20 µM did not increase growth of these mutants. However, the viability of all strains was reduced at 40 µM hemin, which was attributed to the toxicity of heme (Fig. S4). Altogether, these results demonstrate that PPE36 is essential for heme utilization by *M. tuberculosis*. In contrast, *M. tuberculosis* requires PPE62 and Rv0265c for efficient heme utilization but may have other proteins of similar functions.

10.1128/mBio.01720-16.4 FIG S4  Heme-dependent growth of *M*. *tuberculosis* mutants impaired in heme utilization**.** Growth of wt *M. tuberculosis* mc^2^6206 (black) and *ppe36* (red), *ppe62* (green), and *rv0265c* (blue) mutant strains in HdB minimal medium containing increasing concentrations of heme as determined by the microplate alamarBlue assay. Error bars represent standard errors of mean values of biological triplicates. Download FIG S4, TIF file, 1.4 MB. Copyright © 2017 Mitra et al. 2017 Mitra et al. This content is distributed under the terms of the Creative Commons Attribution 4.0 International license.

### PPE36, PPE62, and Rv0265c are novel heme-binding proteins of *M. tuberculosis*.

**FIG 3:**
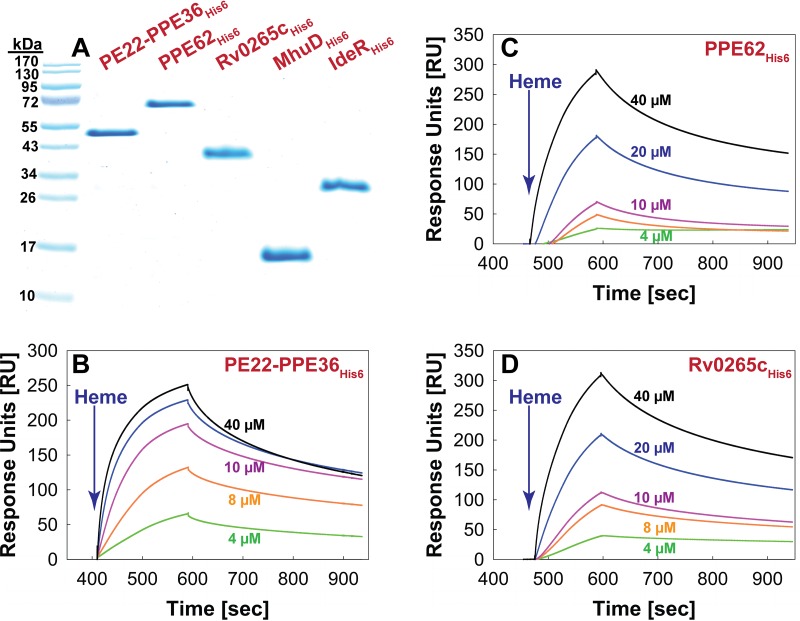
The *M. tuberculosis* proteins PPE36, PPE62, and Rv0265c bind heme. (A) Purified recombinant *M. tuberculosis* proteins PE22-PPE36_His6_, PPE62_His6_, Rv0265c_His6_, MhuD_His6_, and IdeR_His6_. MhuD and IdeR served as positive and negative controls, respectively, for detection of heme binding through SPR (Fig. S5B). (B to D) Heme binding at different concentrations of heme was then determined for PPE36_His6_ (B), PPE62_His6_ (C), and Rv0265c_His6_ (D). Protein-heme interactions were monitored over a 5-min time period and reported as response units (RU).

Possible functional roles of PPE36, PPE62, and Rv0265c include heme binding, uptake, and/or degradation or accessory functions for other proteins directly involved in heme utilization. To examine whether any of these proteins binds heme, we performed surface plasmon resonance (SPR) experiments. For positive and negative controls, we chose the known heme-binding protein MhuD of *M. tuberculosis* (26) and the iron-binding transcriptional regulator IdeR (27), respectively. The hexahistidine-tagged recombinant proteins of MhuD_His6_, IdeR_His6_, PPE62_His6_, and Rv0265c_His6_ were produced in *Escherichia coli* and purified to apparent homogeneity by nickel affinity chromatography (Fig. 3A). No expression was obtained in *E. coli* with a vector containing the *ppe36* gene alone. Since it has been reported that individual *pe* and *ppe* genes did not express well or were expressed in insoluble form (28), we expressed *ppe36* together with its cognate *pe22* gene. The PE22-PPE36_His6_ complex was produced in milligram quantities and was purified by nickel affinity chromatography (Fig. 3A). Then, the control proteins MhuD_His6_ and IdeR_His6_ were immobilized on a nitrilotriacetic acid sensor chip and analyzed in SPR experiments as shown in Fig. S5A. Addition of hemin resulted in a rapid signal increase indicative of hemin binding and a slow dissociation of hemin from MhuD (Fig. S5B). In contrast, IdeR showed only a marginal increase in signal upon hemin addition and an immediate dissociation (Fig. S5B). These results indicated that SPR can be used to detect interactions of proteins with heme. PE22-PPE36_His6_, PPE62_His6_, and Rv0265c_His6_ showed signals similar to that of the MhuD control protein after the addition of hemin. This signal intensity was dependent on the hemin concentration for all proteins (Fig. 3). Kinetic analysis resulted in dissociation constants *K*_*d*_ of 4 × 10^−3^ M, 4 × 10^−4^ M, and 3 × 10^−4^ M for PE22-PPE36_His6_, PPE62_His6_, and Rv0265c_His6_, respectively (Table 1). These results show that PE22-PPE36, PPE62, and Rv0265c bind heme; however, their binding affinities are low compared to most other heme-binding proteins except for IsdB (Table 1). This might be an intrinsic property of these particular *M. tuberculosis* proteins or might result from immobilization of hexahistidine-tagged proteins on a chip in the SPR experiments.

10.1128/mBio.01720-16.5 FIG S5  Analysis of heme binding by proteins through surface plasmon resonance (SPR) spectroscopy. (A) Schematic representation of SPR experiments. His-tagged protein is immobilized on Ni-NTA chip (curve 1). Heme is injected and flown over chip with bound protein (curve 2). Heme-protein interactions are determined at varied heme concentrations (curve 3). All interactions are reported as response units (RU) normalized to those of the protein capture level. (B) MhuD_His6_ and IdeR_His6_ were used as positive- and negative-control proteins for heme binding, respectively, in SPR experiments. Download FIG S5, TIF file, 1.6 MB. Copyright © 2017 Mitra et al. 2017 Mitra et al. This content is distributed under the terms of the Creative Commons Attribution 4.0 International license.

**TABLE 1:** Dissociation constants of known heme-binding proteins

Protein(s)	Heme dissociation constant (M)	Reference
PE22-PPE36	4.0 × 10^−3^	This study
PPE62	4.0 × 10^−4^	This study
Rv0265c	3.0 × 10^−4^	This study
Rv0203	5.4 × 10^−6^	16
MhuD	5.0 × 10^−6^	26
HasA	1.9 × 10^−11^	63
HasR	2 × 10^−7^	60
HtsA	3.1 × 10^−10^	64
IsdA	3.8 × 10^−11^	65
IsdB	2.6 × 10^−4^	66
IsdC	2.6 × 10^−12^	65

### Subcellular localization and surface accessibility of the novel heme-binding proteins of *M. tuberculosis*.

**FIG 4:**
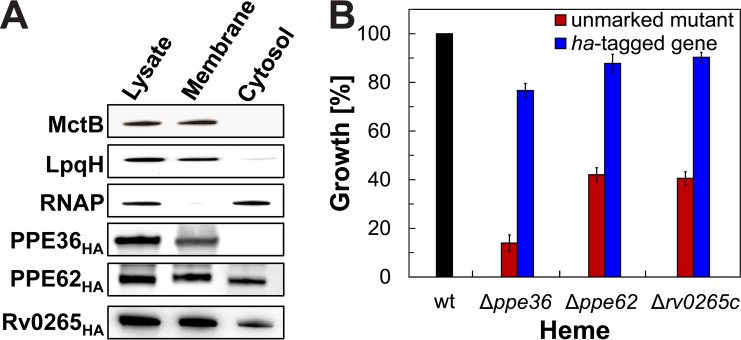
Subcellular localization of the *M. tuberculosis* proteins PPE36, PPE62, and Rv0265c. (A) MctB, LpqH, and RNA polymerase (RNAP) were used as marker proteins for the membrane and cytosolic fraction of *M. tuberculosis*. Monoclonal mouse antibodies were used for detecting MctB, LpqH, and RNAP. PPE62_HA_, Rv0265c_HA_, and PPE36_HA_ were detected using monoclonal mouse antibody against the human influenza hemagglutinin (HA) tag of the proteins. (B) Growth at day 10 in HdB minimal medium with 10 µM hemin and 20 µM of 2,2-dipyridyl determined through microplate alamarBlue assay of wt *M. tuberculosis* mc^2^6206, unmarked mutants, and mutants expressing corresponding HA-tagged genes. Error bars represent standard errors of mean values of biological triplicates.

Next, we determined the subcellular localization of these novel heme-binding proteins in *M. tuberculosis* using vectors encoding C-terminally hemagglutinin (HA)-tagged PPE36 (pML3731), PPE62 (pML3732), or Rv0265c (pML3733) proteins. Cells of the *M. tuberculosis* strains ML2408, ML2409, and ML2410 (Table S3) expressing the *ppe36*_*HA*_, *ppe62*_*HA*_, and *rv0265c_HA_* genes, respectively, were lysed. The membrane fraction was separated from the water-soluble fraction by ultracentrifugation as indicated by MctB and LpqH, which served as positive controls for membrane proteins, and RNA polymerase, which served as a positive control for cytosolic proteins (Fig. 4A). While PPE36_HA_ localized exclusively in the membrane fraction, PPE62_HA_ was detected at similar levels in both membrane and cytosolic fractions. Rv0265c_HA_ localized primarily in the membrane fraction (Fig. 4A). To examine whether the HA tag interfered with protein functions, growth of *M. tuberculosis* mutants expressing the corresponding HA-tagged genes was determined in minimal medium with 10 µM hemin as the iron source. Expression of the HA-tagged genes recovered growth of the mutants to wild-type levels, demonstrating that the HA-tagged proteins were fully functional in heme utilization (Fig. 4B).

**FIG 5:**
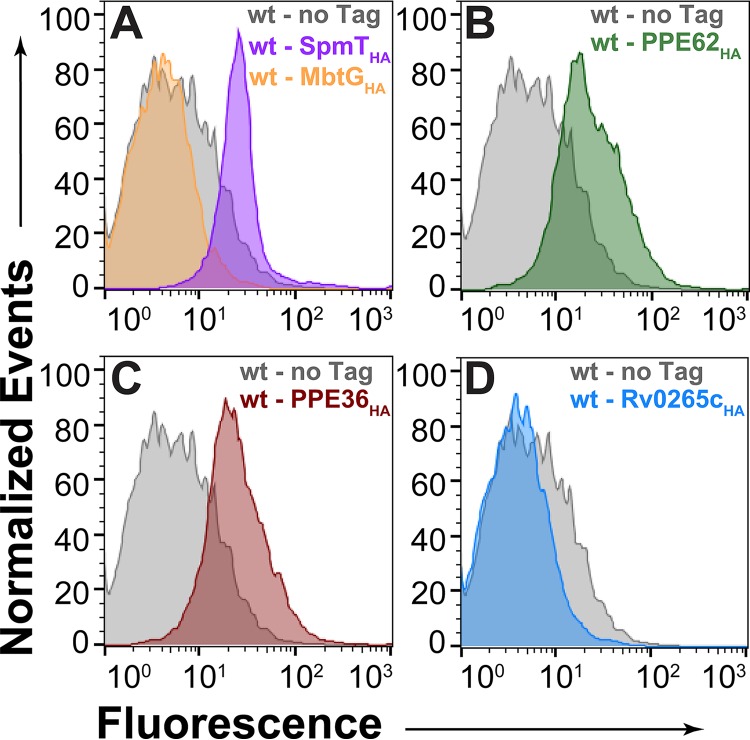
Surface accessibility of *M. tuberculosis* proteins by flow cytometry. Cells of *M. tuberculosis* with integrated vectors ML2437 (empty vector), ML2435 (L5 *attB*::*mbtG_HA_*), ML2436 (L5 *attB*::*rv0888_HA_*) (A), ML2408 (L5 *attB*::*ppe36*_*HA*_) (B), ML2409 (L5 *attB*::*ppe62*_*HA*_) (C), and ML2410 (L5 *attB*::*rv0265c_HA_*) (D) were incubated with monoclonal mouse antibody against the human influenza hemagglutinin (HA) tag followed by detection with anti-mouse FITC-labeled antibody. The mean fluorescence of *M. tuberculosis* cells was measured by flow cytometry and is displayed in histograms.

To distinguish between inner and outer membrane proteins, we used flow cytometry to detect proteins on the cell surface of *M. tuberculosis* as shown before (29, 30). The HA-tagged surface sphingomyelinase SpmT of *M. tuberculosis* was used as a marker protein (30). SpmT_HA_ was detected on the cell surface of *M. tuberculosis* mc^2^6206 in flow cytometry experiments using a monoclonal HA antibody, while only background fluorescence was observed for *M. tuberculosis* containing the empty vector and *mbtG*_*HA*_ (Fig. 5A). This result showed that the C-terminal HA tag of SpmT is surface accessible and indicated that the *M. tuberculosis* cells were mostly intact in this experiment, since the periplasmic MbtG (6) was not detected. *M. tuberculosis* cells producing PPE36_HA_ and PPE62_HA_ exhibited a similar shift in signal as SpmT_HA_ relative to *M. tuberculosis* without any HA-tagged protein (Fig. 5B and C). No increased fluorescence was observed for *M. tuberculosis* cells producing Rv0265c_HA_, indicating that the HA tag of Rv0265c is not surface accessible in these experiments (Fig. 5D). Taken together, these experiments demonstrate that PPE36 and PPE62 are heme-binding cell surface proteins of *M. tuberculosis*.

### PPE36 is not required for virulence of *M. tuberculosis* in mice.

To determine the role of heme utilization by *M. tuberculosis in vivo*, we infected C56BL/6 mice with wt H37Rv, the Δ*ppe36* mutant which cannot utilize heme anymore (Fig. 2B), and the complemented Δ*ppe36* mutant. All strains were grown in Middlebrook 7H9 medium and then cultured in iron-free Middlebrook 7H9 medium for five more days to deplete intracellular iron pools. The similar bacterial loads of lungs and spleens 21 and 42 days after exposure of mice to aerosols with these *M. tuberculosis* strains (Fig. S6) indicate that different iron acquisition pathways of *M. tuberculosis* may substitute for each other during growth and persistence in mice.

10.1128/mBio.01720-16.6 FIG S6  Virulence of the M. tuberculosis Δ*ppe36* mutant during mouse infection. Quantification of bacterial loads in lungs (A) and spleens (B) of C56BL/6 mice infected with *M. tuberculosis* H37Rv, Δ*ppe36* mutant, and the complemented strains. Data are means ± standard deviations (SD) for each group (four mice in each group). Results are representative of two independent experiments. Download FIG S6, TIF file, 1 MB. Copyright © 2017 Mitra et al. 2017 Mitra et al. This content is distributed under the terms of the Creative Commons Attribution 4.0 International license.

## DISCUSSION

### Identification of proteins required for heme utilization by *M. tuberculosis*.

In this study, we identified three proteins of previously unknown functions as important for heme utilization by *M. tuberculosis* by selecting for mutants resistant to the toxic heme analog gallium(III)-porphyrin. Two of these proteins, PPE36 and PPE62, belong to an unusual protein family which is found only in mycobacteria. These PPE proteins share a conserved N-terminal domain of approximately 180 amino acids containing characteristic proline-proline-glutamate motifs and a highly variable C-terminal sequence (31). Growth in a medium with heme as the sole iron source was completely abrogated in the *ppe36* mutant and strongly reduced in the *ppe62* mutant (Fig. 2). These phenotypes are in contrast to the slight growth defect of the *M. tuberculosis* Rv0203 mutant in medium with heme as the sole iron source (16). Rv0203 was shown to bind heme and was proposed to be a secreted hemophore of *M. tuberculosis* (16). The minor role of Rv0203 in heme utilization in *M. tuberculosis* is likely the reason why Rv0203 was not identified in our selection assay using toxic gallium(III)-porphyrin.

### PPE36 is essential for heme utilization by *M. tuberculosis in vitro*.

The PPE36 (Rv2108) protein is essential for heme utilization by *M. tuberculosis* and consists of 243 amino acids comprising the PPE domain of ~180 amino acids and a short C terminus of unique sequence. PPE36 belongs to the PPE sublineage III, comprising small proteins such as PPE41 (31). *ppe41* and many other *ppe* genes are expressed only as folded proteins in a complex with their cognate PE proteins (28). Similarly, the *pe22* (*rv2107*) gene is located upstream of the *ppe36* (*rv2108*) gene and is required for expression of *ppe36* in *E. coli*. On the basis of these observations, it has been proposed that these PE and PPE proteins are exported as folded heterodimers in *M. tuberculosis* (28, 32) with their final localization either at the cell surface of *M. tuberculosis* and/or in the culture medium (33). Flow cytometry experiments showed that PPE36 is a surface-accessible protein consistent with previous observations for several other PPE proteins (34, 35). PPE36 is exclusively associated with membranes in subcellular fractionation experiments in contrast to PPE62 and Rv0265c. However, neither PPE36 nor any other small PPE protein have a predicted transmembrane helix. Furthermore, the structure of the similar PPE41/PE25 complex also does not show any known hydrophobic domain (28, 32). Thus, it is unclear how PPE36 is anchored to the cell surface. Our observation that PPE36 is completely associated with membranes when *M. tuberculosis* was grown in a shaking culture in the presence of detergents appears to exclude the possibility that PPE36 is attached to the polysaccharide capsule as proposed by Ates et al. for other PPE proteins (33). It is also unknown whether PPE36 requires PE22 for heme binding and/or uptake. PPE17 is a PPE protein that does not require its cognate PE proteins for stability and surface localization (35). Further experiments are needed to examine whether this is the case for PPE36.

### PPE62 is required for efficient heme utilization by *M. tuberculosis* and has a different function than PPE36.

PPE62 (Rv3533c) consists of 582 amino acids and belongs to the PPE-major polymorphic tandem repeat (MPTR) family (31). Heme utilization by *M. tuberculosis* was strongly reduced by deletion of *ppe62*, indicating that PPE62 is required for efficient heme utilization but not essential (Fig. 2C). Except for the conserved N-terminal PPE domain, PPE62 does not share any sequence similarity with PPE36. *ppe62* expression does not complement the *Δppe36* mutant, demonstrating that these proteins have different functions in heme utilization. Interestingly, secondary structure prediction for PPE62 indicated that the tandem repeats may form a right-handed parallel β-helix consisting of three parallel β-sheets (see Fig. S7 in the supplemental material). This predicted structure has a striking resemblance to HxuA, which is the cell surface receptor of hemopexin in *Haemophilus influenzae*. The crystal structure of HxuA shows a right-handed β-helix with three parallel β-sheets that are involved in binding and release of heme from host hemopexin (36). Whether the predicted β-helix of PPE62 functions in binding and release of heme in a similar manner as in HxuA remains to be determined. Similar to PPE36, flow cytometry and subcellular fractionation experiments showed that PPE62 is a surface-accessible membrane protein. Although the molecular basis for membrane localization of PPE62 is unknown as for all other PPE proteins, it is interesting that 16 out of 20 PPE-MPTR proteins have been predicted to be outer membrane proteins based on secondary structure predictions of amphiphilic β-strands that may form a β-barrel with a hydrophobic surface (37, 38). This includes the surface proteins PPE62 and PPE34 (34), but not PPE36 and PPE17 (35), which belong to other PPE subfamilies.

10.1128/mBio.01720-16.7 FIG S7  Predicted secondary structure of PPE62. (A) Schematic representation of predicted domains of PPE62 using RaptorX. The conserved α-helical N-terminal PPE domain (red), right-handed β-helix comprised of three parallel β-sheets (blue, green, and cyan), and an unstructured domain (black) are shown. (B) Side view of the predicted PPE62 β-helix, which is structurally similar to HxuA of *Haemophilus influenzae* (36, 67). (C) Top view of the three parallel β-sheets (blue, green, and cyan) forming the triangular β-helix structure as similarly seen in HxuA. Download FIG S7, JPG file, 1.2 MB. Copyright © 2017 Mitra et al. 2017 Mitra et al. This content is distributed under the terms of the Creative Commons Attribution 4.0 International license.

### Rv0265c is required for efficient heme utilization by *M. tuberculosis*.

**FIG 6:**
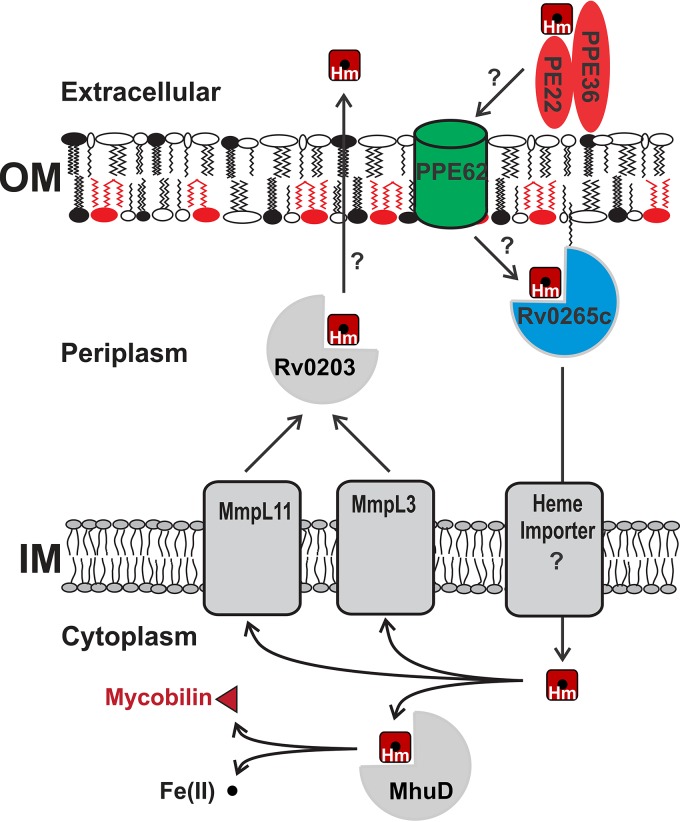
Model of heme uptake by *M. tuberculosis*. PPE36 is a membrane-bound surface-accessible protein that is essential for heme utilization by *M. tuberculosis*. The PPE36/PE22 complex binds heme (Hm), but it is unknown whether complex formation is required for heme binding. PPE62 is a surface-accessible, heme-binding protein anchored in the outer membrane (OM). Rv0265c is a periplasmic heme-binding protein that might transfer heme to a yet unknown transporter in the inner membrane (IM). Cytoplasmic heme is degraded to mycobilin by the MhuD oxygenase, thereby releasing iron (small black circle) (20). MmpL3 and MmpL11 are IM efflux pumps which are capable of heme transfer to Rv0203 (46).

The ability of the *M. tuberculosis rv0265c* mutant to grow in medium with heme as the sole iron source is reduced compared to the parent strain (Fig. 2D), identifying Rv0265c as the third *M. tuberculosis* protein involved in heme utilization. However, the growth phenotype of the *M. tuberculosis rv0265c* deletion mutant is weaker than those of the *ppe36* and *ppe62* mutants, indicating that Rv0265c has a partially redundant role in heme utilization by *M. tuberculosis*. The *rv0265c* deletion mutant has a growth defect even in medium with ferric citrate as the sole iron source (Fig. 2A), indicating that Rv0265c may have functions in addition to its role in heme utilization. Rv0265c consists of 330 amino acids and has a signal peptide targeting the protein to the twin-arginine translocation (TAT) pathway, which is utilized for export of folded proteins (39). Interestingly, Rv0265c also contains a cysteine at the N terminus after the predicted signal peptidase cleavage site, indicating that Rv0265c is a lipoprotein (40). This is consistent with our observation that Rv0265c is primarily localized in the membrane fraction in subcellular fractionation experiments with *M. tuberculosis* (Fig. 4). In *E. coli*, lipoproteins with an aspartate residue in position +2 after the cysteine are localized on the outer leaflet of the inner membrane, whereas all other lipoproteins are directed to the outer membrane (40). Rv0265c does not have an aspartate residue following the N-terminal cysteine of the mature protein, which may indicate targeting to the outer membrane. However, it is not clear how lipoproteins are sorted in mycobacteria and whether a system similar to the Lol system of *E. coli* exists in *M. tuberculosis*. The crystal structure of Rv0265c was solved (PDB identifier: 4PM4) and shows significant structural similarities to HmuT, the periplasmic component of the heme uptake pathway in *Yersinia pestis* (41). Taken together, a model in which Rv0265c is located in the inner leaflet of the outer membrane is consistent with the results of this study and our bioinformatic analysis (Fig. 6).

### A new model for heme acquisition by *M. tuberculosis*.

Growth of *M. tuberculosis* is strongly reduced using heme as an iron source versus the siderophore-mediated utilization of ferric citrate (26 and 11 days, respectively [Fig. 2]). This result is consistent with the previous observation that growth of *M. tuberculosis* on hemin requires 1,000-fold-higher concentrations compared to ferric cMBT as an iron source (7). However, heme acquisition by *M. tuberculosis* is probably important due to the abundance of heme-bound iron compared to other iron sources in the human body (42). The findings of this study substantially expand our understanding of the mechanism by which *M. tuberculosis* utilizes heme as an iron source as depicted in a revised model (Fig. 6). PPE36 may capture heme on the cell surface of *M. tuberculosis* (Fig. 6). It is unknown whether complex formation with PE22 is required for heme binding and/or surface attachment of PPE36. PPE62 is predicted to be an outer membrane protein (37, 38) and may function as a heme receptor similar to HasR of *Serratia marcescens* (43). Since PPE62 is not essential for heme utilization, it is likely that *M. tuberculosis* produces at least one additional protein which can partially substitute for PPE62. Ultimately, an atomic structure is required to provide final evidence whether PPE62 forms a membrane-spanning hydrophobic domain such as those found in other mycobacterial outer membrane proteins (44). Our observation that PPE36 and PPE62 are heme-binding surface proteins suggests that they are unlikely to be involved in heme detoxification which is mainly dependent on efflux by inner membrane transporters and/or sequestration by cytoplasmic and periplasmic proteins in other bacteria (45). Rv0265c is an exported, heme-binding protein of *M. tuberculosis* whose structure is similar to that of HmuT, a periplasmic heme-binding protein of *Y. pestis* (41). It is unclear in which membrane Rv0265c is anchored. Interestingly, none of the genes identified in this study is regulated by iron or by the major iron regulator IdeR (27), indicating that expression of these genes might be specifically induced by heme. We propose that PPE36 and PPE62 are involved in heme translocation across the outer membrane. Heme uptake across the inner membrane is mediated by an unknown transporter. Then, the heme oxygenase MhuD degrades the porphyrin ring and releases iron from heme without generating carbon monoxide (20). Rv0203 is a heme-binding protein (16) which transfers heme to water-soluble domains of the inner membrane proteins MmpL3 and MmpL11 (46) in the periplasm of *M. tuberculosis* (47). MmpL proteins are efflux pumps (48, 49) that export hydrophobic molecules such as lipids (50–52), azoles (53), and siderophores (6) out of the cell. Thus, it is difficult to reconcile heme uptake, as proposed for MmpL3 and MmpL11 (16), with the opposite transport directionality of MmpL proteins. Hence, we propose that MmpL3 and MmpL11 might be involved in heme efflux to protect *M. tuberculosis* from the toxicity of excess heme as described for other bacterial pathogens (45). Redundancy of MmpL3 and MmpL11 is also consistent with the slight growth defects of the *mmpL3* and *mmpL11* mutants in the presence of heme (16).

### Conclusions.

PPE36 and PPE62 are, to our knowledge, the first PPE proteins of *M. tuberculosis* with functions in nutrient acquisition. Previously, PPE proteins were thought to be involved in antigenic variation and immune evasion (54, 55). While our study identified three novel proteins involved in heme utilization by *M. tuberculosis*, their molecular functions and how these proteins interact with each other are unknown. Further, crucial components of the heme uptake system are still missing (Fig. 6). The 1,000-fold-lower efficiency of iron acquisition from heme compared to ferric siderophore uptake might be balanced by the much higher abundance of heme in the human body (11). Taken together, the emerging model indicates that *M. tuberculosis* has evolved a heme uptake system different from those in other bacteria (56–60).

## MATERIALS AND METHODS

### Bacterial strains, media, and growth conditions.

Virulent *M. tuberculosis* H37Rv and its derivative strains were grown in Middlebrook liquid 7H9 or solid 7H10 medium supplemented with 10% oleic acid-albumin-dextrose-catalase (OADC) (8.5 g/liter NaCl, 20 g/liter dextrose, 50 g/liter bovine albumin fraction V, 0.03 g/liter catalase, 0.6 ml/liter oleic acid). Avirulent *M. tuberculosis* mc^2^6206 and its derivative strains were grown in Middlebrook liquid 7H9 or solid 7H10 medium supplemented with 0.5% glycerol, 10% albumin-dextrose-salt (ADS) (8.5 g/liter NaCl, 20 g/liter dextrose, 50 g/liter bovine albumin fraction V), 0.2% Casamino Acids, 24 μg/ml pantothenate, and 50 µg/ml l-leucine. *Escherichia coli* DH5α was grown in LB medium containing appropriate antibiotics at 37°C with shaking at 200 rpm. The following antibiotics were used when required: ampicillin (Amp) at 100 μg/ml for *E. coli*, kanamycin (Kan) at 30 μg/ml for mycobacteria and 50 μg/ml for *E. coli*, and hygromycin (Hyg) at 200 μg/ml for *E. coli* and 50 μg/ml for mycobacteria.

### Synthesis of gallium(III)-porphyrin.

Gallium(III)-porphyrin (Ga-PIX) was synthesized as previously described (24). Briefly, 2.4 g of gallium chloride (GaCl_3_), 5.3 g of protoporphyrin IX (PIX), and 5.0 g of sodium acetate were added to 400 ml of acetic acid, refluxed for 16 h, and then cooled on ice to allow crystallization. All excess acetic acid was removed by evaporation, and excess GaCl_3_ and sodium acetate were dissolved in 200 ml water and removed, leaving 30 mg of a brown precipitate containing the insoluble Ga-PIX and any unreacted PIX. The dried precipitate was dissolved in 10 ml dimethyl sulfoxide (DMSO). Ga-PIX and PIX were excited with light with a wavelength of 410 nm, and the fluorescence of synthesized Ga-PIX (at 585 nm) was compared to that of PIX (at 610 nm) and quantified. Ga-PIX and PIX were also characterized by thin-layer chromatography (TLC) on silica gel plates using a mobile phase consisting of methanol and water in a 3:1 volume ratio. PIX, hemin, and Ga-PIX were visualized using UV light.

### **Toxicity of gallium(III)-porphyrin and precursors for** M. tuberculosis**.**

The MICs of GaCl_3_, PIX, and Ga-PIX were determined against avirulent *M. tuberculosis* mc^2^6206. Cultures were grown to log phase in Middlebrook 7H9 medium, washed in sterile phosphate-buffered saline (PBS) (137 mM NaCl, 2.7 mM KCl, 10 mM Na_2_HPO_4_, 1.8 mM KH_2_PO_4_) with 0.02% Tyloxapol (PBS–Tyloxapol) and then depleted of iron for three or four generations in iron-free Middlebrook 7H9 medium. In 96-well plates, washed *M. tuberculosis* cells were inoculated at an optical density at 600 nm (OD_600_) of 0.05 into iron-free Middlebrook 7H9 medium containing 2 µM ferric carboxymycobactin (cMBT) as the iron source and various concentrations of GaCl_3_, PIX, or Ga-PIX. After 5 or 6 days of growth, the MIC_90_s for all compounds were determined using an alamarBlue assay as described previously (61).

### Targeted gene deletion in M. tuberculosis.

A schematic representation of targeted gene deletion is shown in Fig. S2 in the supplemental material. For all genes, upstream (U) and downstream (D) sequences were amplified using corresponding primer pairs UF/SpeI-UR/SwaI (F stands for forward, and R stands for reverse) and DF/PacI-DR/NsiI (Table S2), respectively, and cloned into pML2424. pML3715, pML3723, and pML3726 (Table S1) were transformed into avirulent *M. tuberculosis* mc^2^6206 for deletion of *ppe36*, *ppe62*, and Rv0265 genes, respectively. Transformants were selected at 37°C on Middlebrook 7H10 medium supplemented with hygromycin (Hyg) and visually validated by the presence of both green fluorescent protein (GFP) and red fluorescent protein (RFP) fluorescence. Liquid cultures of transformants were then plated on Middlebrook 7H10 medium supplemented with Hyg containing 2% sucrose at 40°C for selection of double crossovers. Putative double crossovers were visually analyzed for the presence of only GFP, and gene deletion was validated by PCR (Fig. S2). For excision of the *loxP*-flanked *gfp*^2*+*^*_m_-hyg* cassette, pML2714 expressing Cre recombinase was transformed into marked mutants, and unmarked mutants were selected on Middlebrook 7H10 medium supplemented with Kan at 37°C. Putative unmarked mutants were first visually validated through the absence of GFP fluorescence and then through PCR (primers [Table S2]) and loss of growth on hygromycin. Unmarked mutants of *ppe36*, *ppe62*, and *rv0265c* gene were designated ML2411, ML2412, and ML2413, respectively. All strains are described in Table S3.

10.1128/mBio.01720-16.8 TABLE S1  Plasmids used in this study. Download TABLE S1, DOCX file, 0.03 MB. Copyright © 2017 Mitra et al. 2017 Mitra et al. This content is distributed under the terms of the Creative Commons Attribution 4.0 International license.

10.1128/mBio.01720-16.9 TABLE S2  Primers used in this study. Download TABLE S2, DOCX file, 0.03 MB. Copyright © 2017 Mitra et al. 2017 Mitra et al. This content is distributed under the terms of the Creative Commons Attribution 4.0 International license.

10.1128/mBio.01720-16.10 TABLE S3  Strains used in this study. Download TABLE S3, DOCX file, 0.03 MB. Copyright © 2017 Mitra et al. 2017 Mitra et al. This content is distributed under the terms of the Creative Commons Attribution 4.0 International license.

### Growth experiments for determining iron utilization.

Strains were first grown in Middlebrook 7H9 medium, then washed in sterile PBS with 0.02% Tyloxapol, and iron depleted for 3 or 4 generations in iron-free Middlebrook 7H9 medium. Strains were then inoculated into HdB minimal medium (62) containing either 10 µM hemin or 10 µM ammonium ferric citrate as the sole iron source. To prevent growth from traces of iron, 20 µM iron chelator 2,2-dipyridyl (DIP) was added to medium containing heme.

### Construction of expression vectors and strains for mycobacterial genes.

The open reading frames (ORFs) of *ppe36*, *ppe62*, and *rv0265c* were amplified using corresponding primers, 016Clone/F and 016Clone/R (Table S2), and cloned into pMN016 to construct pML3716, pML3724, and pML3727, respectively. pML3716, pML3724, and pML3727 were transformed into strains ML2411, ML2412, and ML2413 to create strains ML2414, ML2415, and ML2416, respectively. All of these strains were used in subsequent growth experiments for determining heme utilization. For hemagglutinin (HA)-tagged expression of *M. tuberculosis* proteins, ORFs of *ppe36*, *ppe62*, and *rv0265c* were amplified using the corresponding primers, primers HA/F and HA/R (Table S2), and cloned into pML1391 to construct pML3731, pML3732, and pML3733, respectively. pML3731, pML3732, and pML3733 were transformed into avirulent *M. tuberculosis* mc^2^6206 to create strains ML2408, ML2409, and ML2410, respectively, which were then used in subcellular localization and surface detection experiments. pML1828 and pML2109 expressing MbtG_HA_ and Rv0888_HA_ were transformed into avirulent *M. tuberculosis* mc^2^6206 to create strains ML2435 and ML2436, respectively, which were negative and positive controls for surface detection experiments.

### **Subcellular fractionation of** M. tuberculosis**.**

Strains ML2408, ML2409, and ML2410 were grown in Middlebrook 7H9 supplemented with ADS and Hyg to an OD_600_ of 2. Cells were harvested by centrifugation, washed twice with PBS containing 1 mM phenylmethylsulfonyl (PMSF) and lysed by sonication (20 min, 12-W output power). Cell debris was removed from the lysate by centrifugation at 3,200 × *g* for 10 min at 4°C, and the clear lysate (L) was centrifuged at 100,000 × *g* for 1 h at 4°C. The supernatant (C1) was transferred to a separate tube, and the pellet was resuspended in the same volume of PBS as C1 and designated M1. Both C1 and M1 fractions were centrifuged at 100,000 × *g* for 1 h at 4°C. The supernatant containing the cytosolic fraction was transferred to a new tube and labeled C2, and the membrane pellet fraction was resuspended in the same volume of PBS as that used for C2 and designated M2. HA-tagged proteins in L, M2, and C2 fractions were detected in Western blots using a horseradish peroxidase-coupled mouse antibody against HA (catalog no. H6533; Sigma). The membrane control proteins MctB and LpqH and the cytosolic control protein RNA polymerase were detected by monoclonal mouse antibodies. Blots were developed using enhanced chemiluminescence (ECL) Western blotting substrate (Pierce), and luminescence was visualized using LabWorks (UVP, Inc.) imaging system.

### **Surface detection of proteins in** M. tuberculosis **by flow cytometry.**

Strains ML2408 (PPE36_HA_), ML2409 (PPE62_HA_), ML2410 (Rv0265_HA_), ML2435 (MbtG_HA_), and ML2408 (Rv0888_HA_) were used for surface detection experiments. Cultures were grown to mid-log phase and fixed with 4% paraformaldehyde for 30 min at room temperature. The cells were washed three times with PBS–Tyloxapol (0.02%) and incubated with monoclonal rabbit anti-HA antibody at a 1:1 dilution for 2 h for bacterial surface staining. The cells were then washed three times with PBS–Tyloxapol and then stained with fluorescein isothiocyanate (FITC)-labeled anti-rabbit antibodies at a dilution of 1:100 for 2 h. The cells were again washed three times with PBS–Tyloxapol and analyzed via flow cytometry. Surface-accessible proteins were quantified by measuring fluorescence and displayed as histograms.

### Construction of protein overexpression vectors and protein purification.

The ORFs of all target genes were amplified using the corresponding primers, 21aClone/F and 21aClone/R (Table S2), and cloned into pET-21a+. *ppe36* (*rv2108*) was cloned with its cognate *pe22* gene, *rv2107*, to create the expression plasmid pML3720. Finally, the *ppe62*, *ideR*, *mhuD*, and *rv0265c* genes, excluding its signal peptide, were all cloned into pET-21a to create pML3730, pML3754, pML3755, and pML3729, respectively. Transformants in *E. coli* BL21(DE3) were selected on LB agar plates containing Amp. Starter cultures of strains were inoculated into 500 ml of fresh LB with Amp and grown to an OD_600_ of 0.3. Protein expression was induced with 1 mM isopropyl-β-d-thiogalactopyranoside (IPTG) at 16°C for 14 h. Cells were harvested by centrifugation and lysed by sonication in ice-cold lysis buffer (20 mM Tris, 300 mM NaCl, 1 mM PMSF [pH 7.4]). Cell lysate was clarified by centrifugation, and the supernatant was loaded on to activated nickel resin and bound overnight at 4°C with shaking. Protein-loaded resin was washed three times with wash buffer (lysis buffer with 25 mM imidazole), and target protein was eluted with elution buffer (lysis buffer with 250 mM imidazole). Purified recombinant proteins were quantified by the Bradford assay. PE22-PPE36_His_ and PPE62_His_ were purified in buffers containing 0.5% (vol/vol) *n*-octyl-polyoxyethylene (OPOE).

### Surface plasmon resonance spectroscopy.

Heme binding by recombinant MhuD_His6_, IdeR_His6_, PPE36_His6_, PPE62_His6_, and Rv0265_His6_ was determined by surface plasmon resonance (SPR) spectroscopy using a Biacore T200 molecular interaction system (GE Healthcare). The SPR experimental scheme is depicted in Fig. S5A. HBS-EP (10 mM HEPES, 150 mM NaCl, 3 mM EDTA, and 0.005% polysorbate 20 [pH 7.4]) was used as the running buffer for the immobilization and kinetic studies. In a Series S Sensor Chip NTA (Biacore), recombinant protein (ligand) was immobilized onto a flow cell at a flow rate of 5 µl/min for 5 min until a ligand density of ~200 response units (RU) was obtained. Following ligand capture, heme (analyte) at different concentrations (diluted in running buffer) was injected into the flow cell at 10 µl/min for 2 min to observe association, and then dissociation was allowed for 5 min. As a control, a reference flow cell without any bound nickel was used before ligand capture, with all the following same steps as the active flow cell. All RU values were normalized to those of the protein capture level, and the binding response was reported as the difference of RU values between the active flow cell and the control flow cell. Within the Biacore evaluation software, a 1:1 binding model was used to fit the binding response curves and dissociation constant *K*_*d*_ was calculated for all target proteins.

### Mouse infections.

Female C57BL/6 mice (Jackson Laboratory) were infected using an inhalation exposure system (Glas-Col) with *M. tuberculosis* grown to mid-log phase in iron-depleted Middlebrook 7H9 medium to deliver approximately 100 bacilli per mouse. Lungs and spleens were harvested at the indicated time points and homogenized in PBS, serially diluted, and cultured on Middlebrook 7H10 agar to quantify CFU. All animal experiments were performed following National Institutes of Health guidelines for housing and care of laboratory animals and performed in accordance with institutional regulations. The protocol was reviewed and approved by the Institutional Animal Care and Use Committee of Weill Cornell Medical College.
