# Supplementary figures and images for: PPE Surface Proteins Are Required for Heme Utilization by Mycobacterium tuberculosis
Source: mBio. 2017 Jan 24;8(1):e01720-16. doi: 10.1128/mBio.01720-16 (PMC5263243; doi:10.1128/mBio.01720-16)

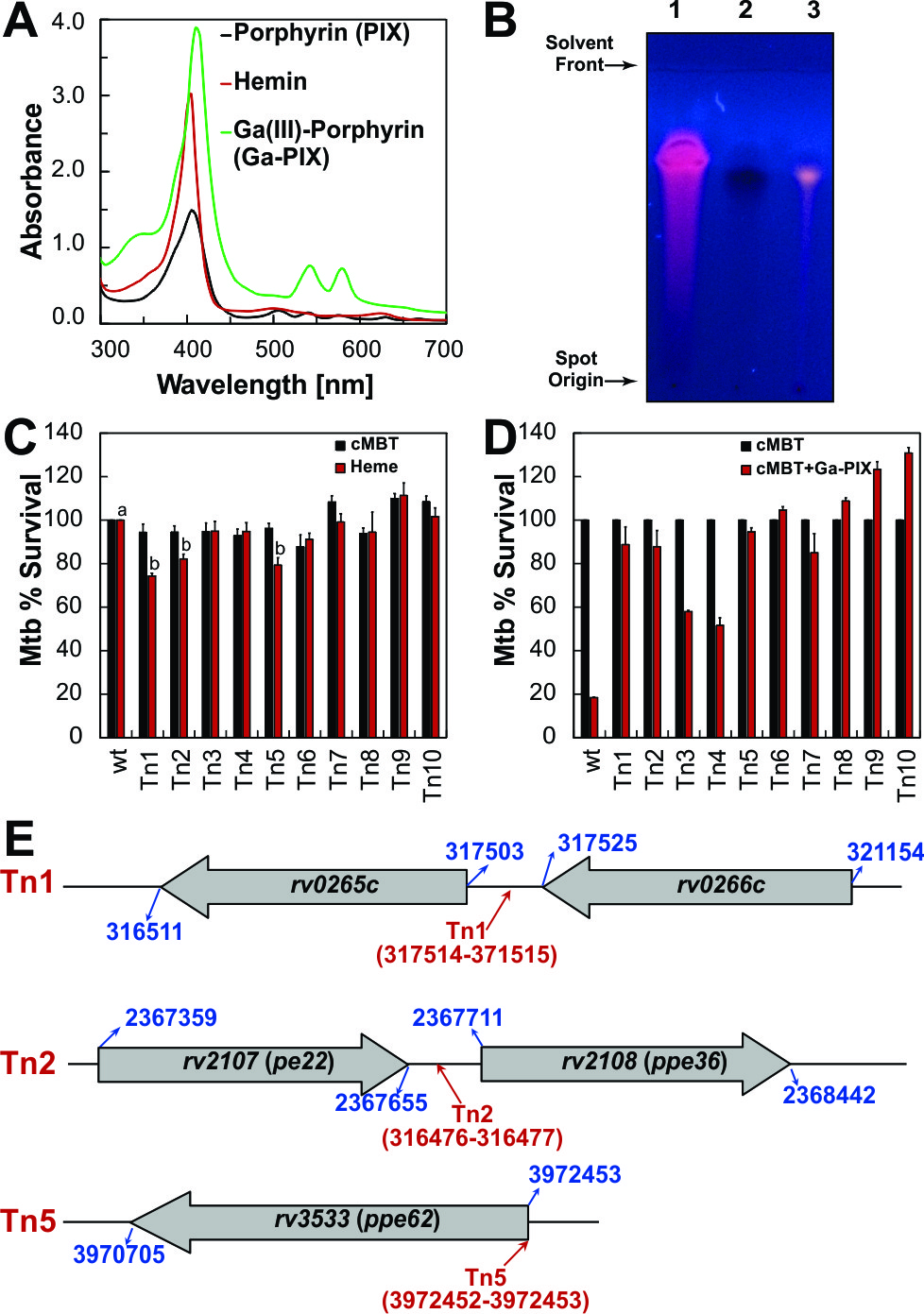

Supplement: FIG S1 [file mbo001173164sf1.jpg]

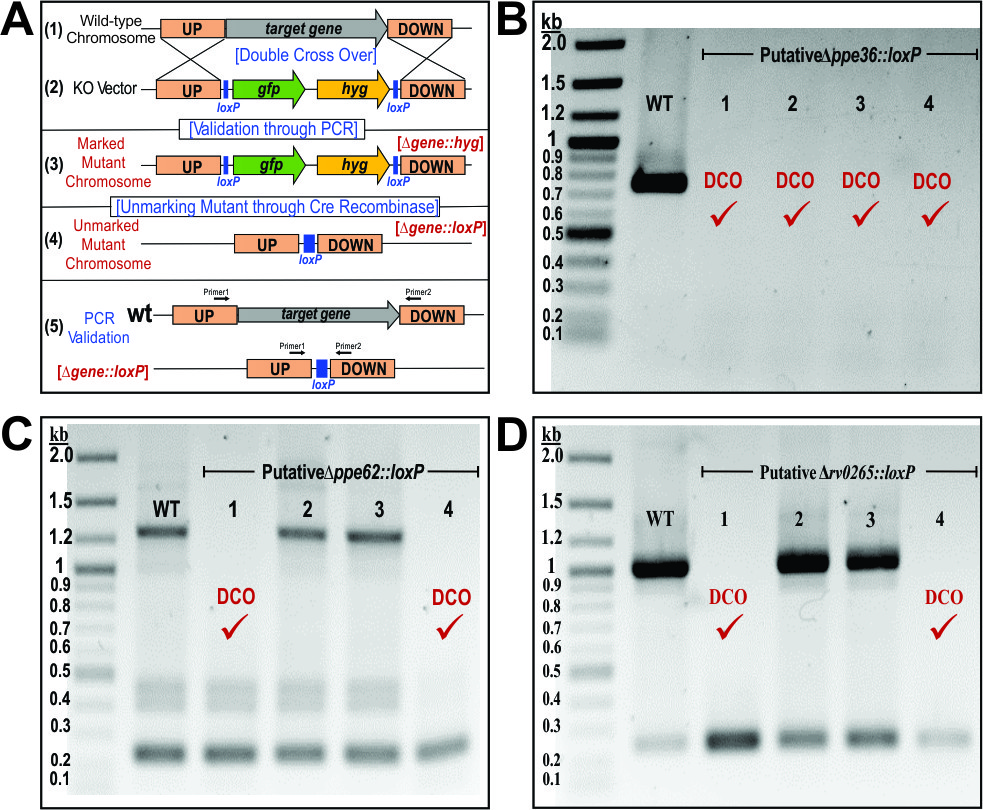

Supplement: FIG S2 [file mbo001173164sf2.jpg]

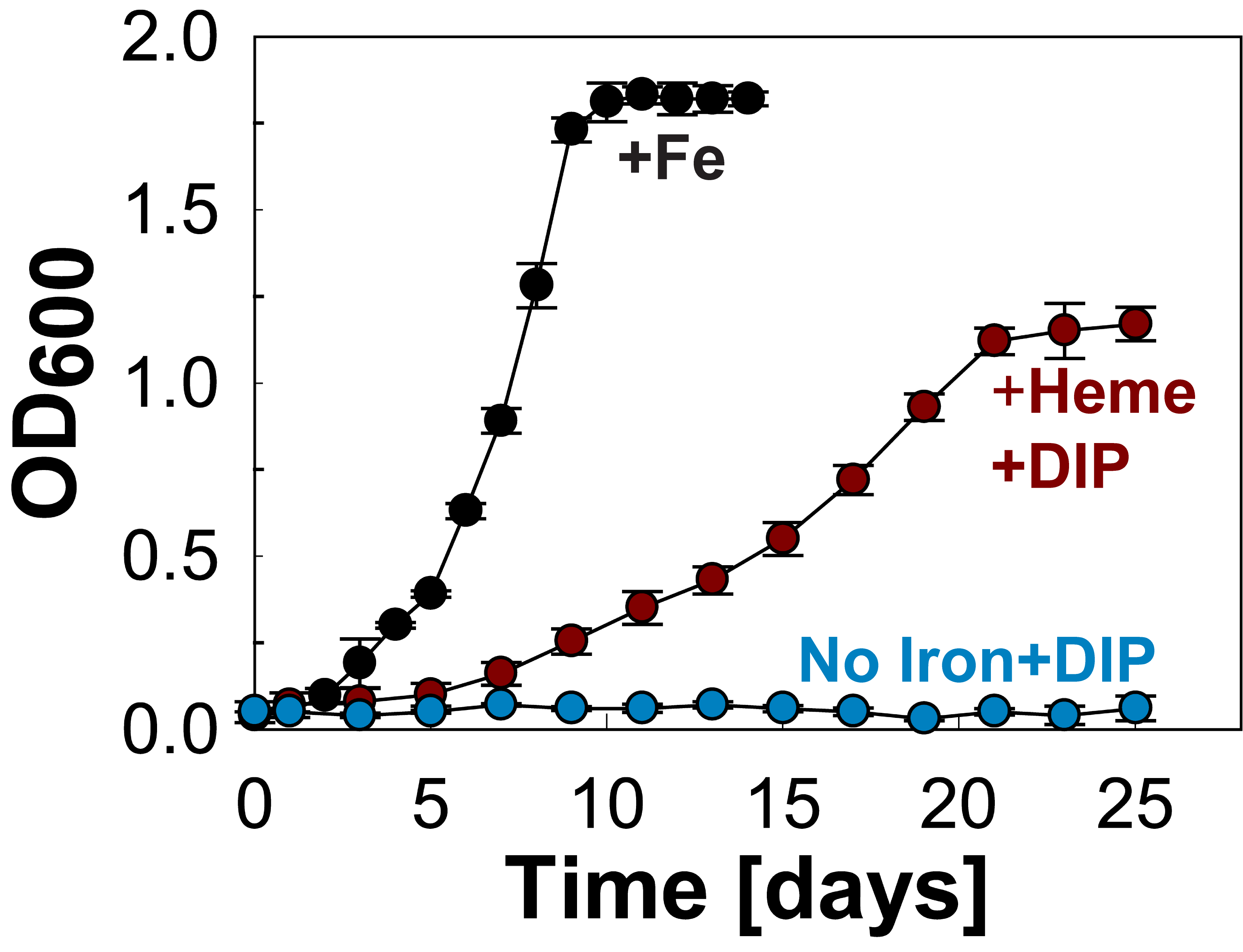

Supplement: FIG S3 [file mbo001173164sf3.tif]

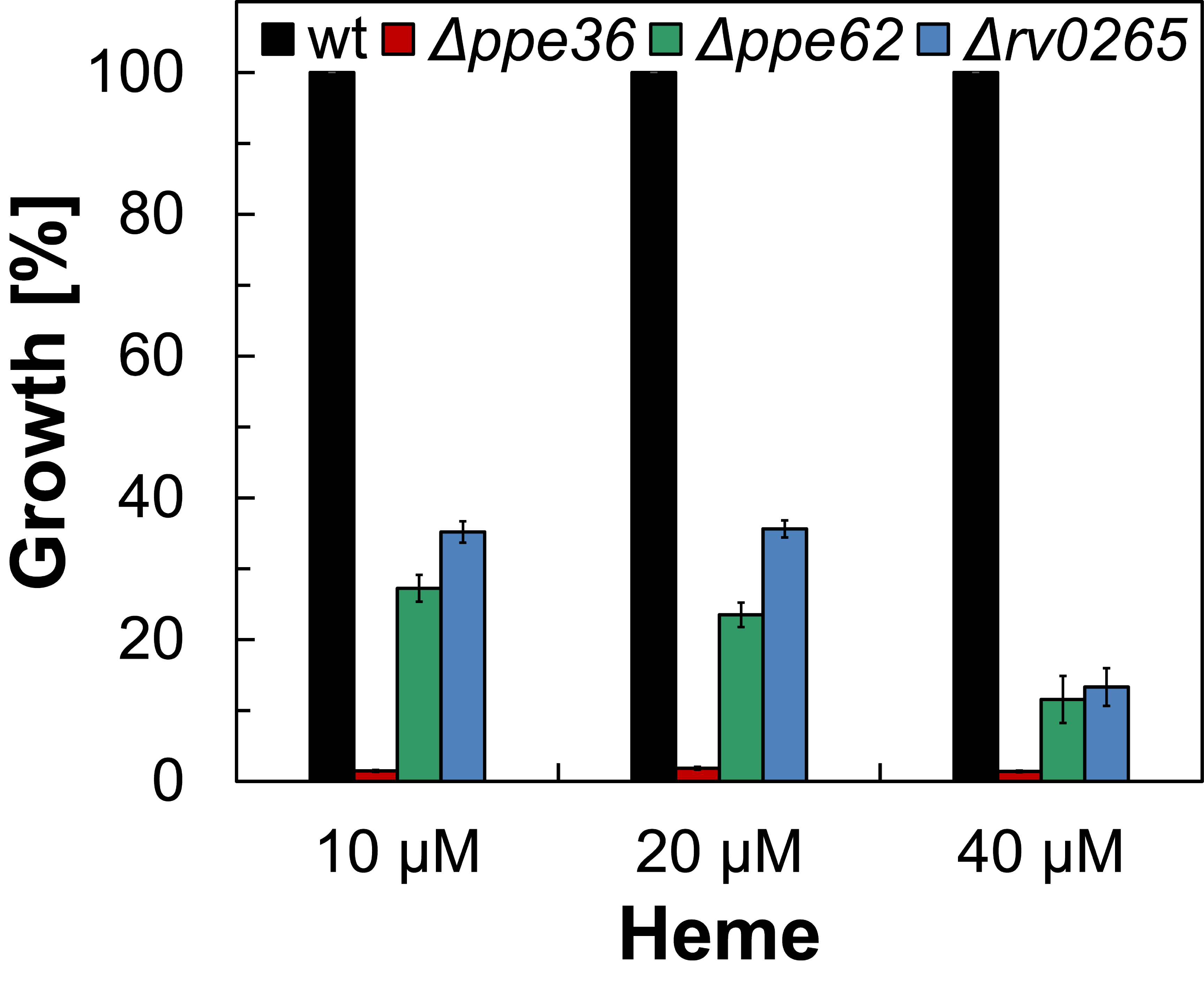

Supplement: FIG S4 [file mbo001173164sf4.tif]

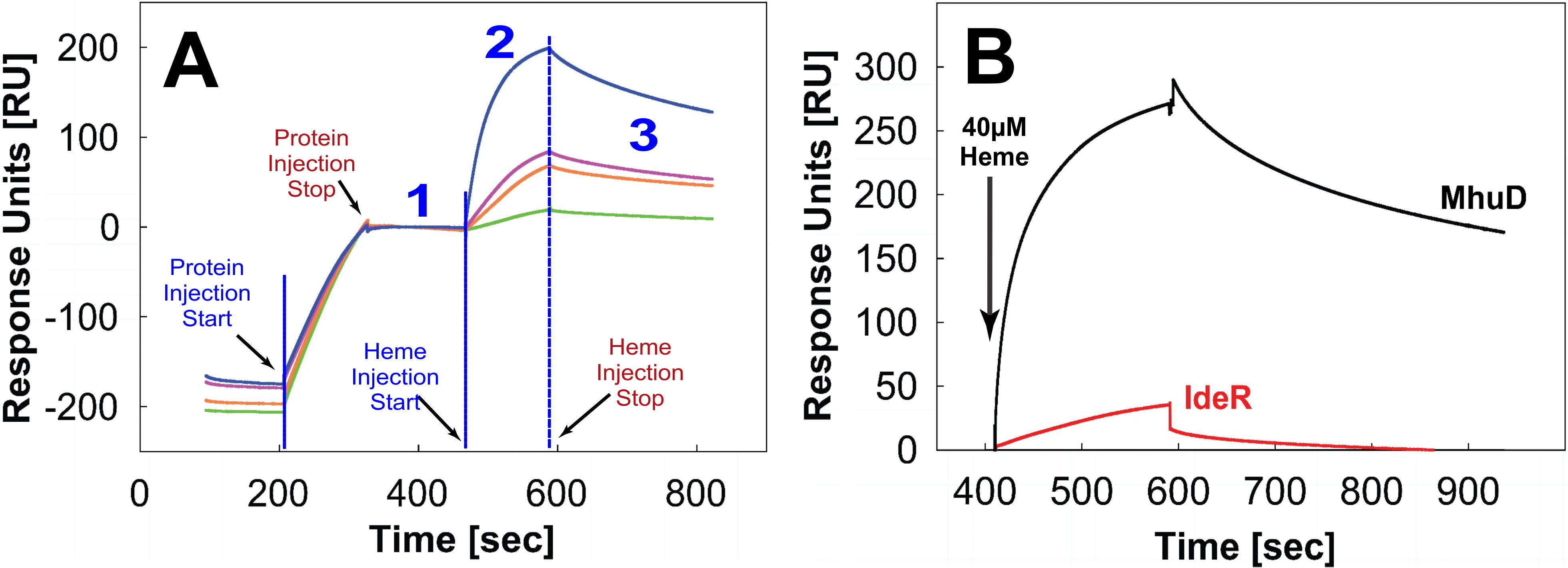

Supplement: FIG S5 [file mbo001173164sf5.tif]

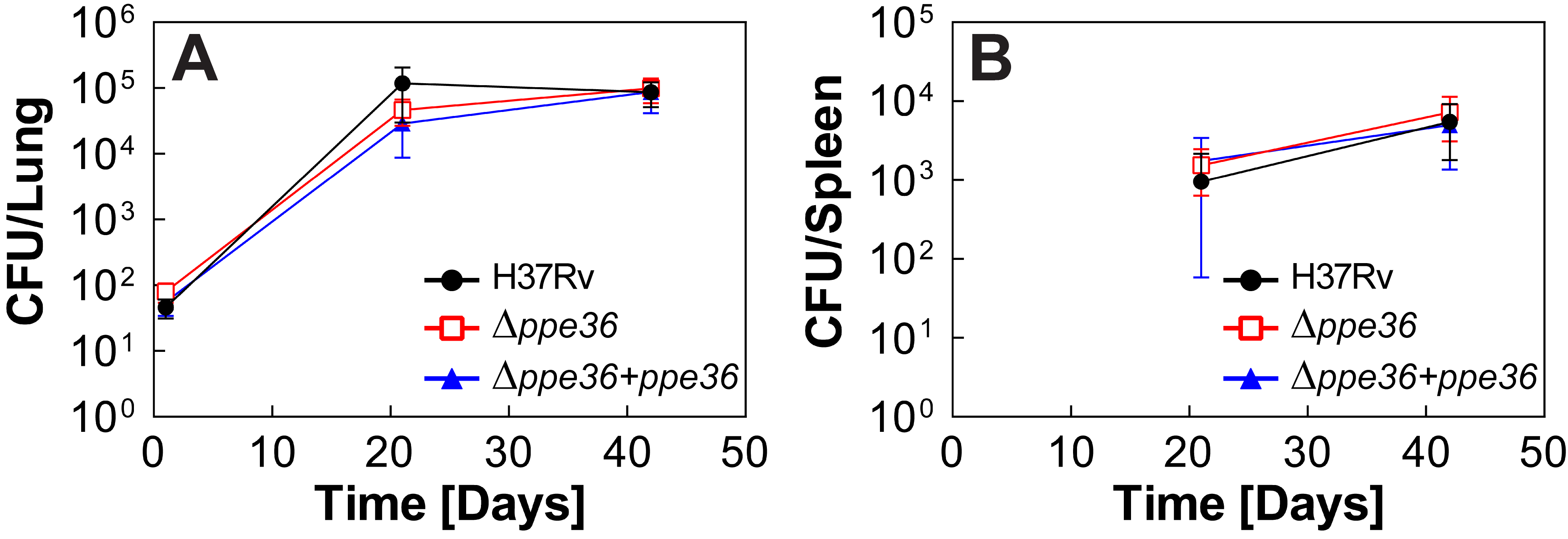

Supplement: FIG S6 [file mbo001173164sf6.tif]

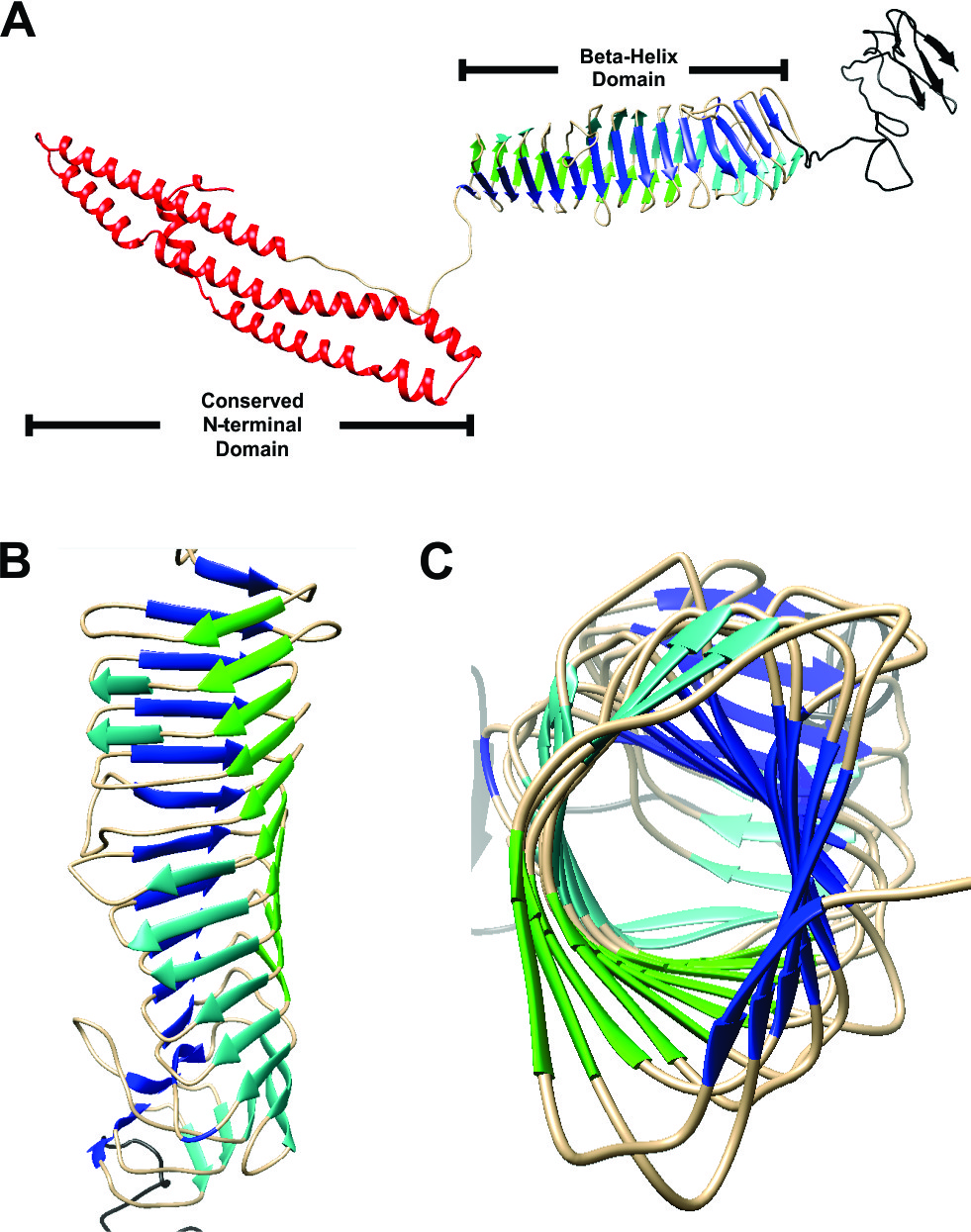

Supplement: FIG S7 [file mbo001173164sf7.jpg]
