# Supplementary material for: PPE Surface Proteins Are Required for Heme Utilization by Mycobacterium tuberculosis
Source: mBio. 2017 Jan 24;8(1):e01720-16. doi: 10.1128/mBio.01720-16 (PMC5263243; doi:10.1128/mBio.01720-16)
Supplement: TABLE S1 [file mbo001173164st8.docx]

| **Name** | **Description** | **Abx Marker** |
| --- | --- | --- |
| ***Parent Vectors*** | |  |
| pML2424 | parent vector for construction of KOs in Mycobacteria by homologues recombination; HygR | Hyg |
| pET21a+ | Plasmid used for His-tagged protein purification | Amp |
| pMN016 | cloning vector for expression of mycobacterial genes under strong **psmyc** promoter using PacI-HindIII restriction sites | Hyg |
| pML1391 | cloning vector for expression of C-terminal HA-His-tagged mycobacterial genes under strong psmyc promoter using SphI-EcoRV restriction sites | Hyg |
| pML2714 | *Cre* recombinase vector for excision of *gfp-hyg* cassette utilizing *loxP* sites | Kan |
| ***Knockout Vectors*** | |  |
| pML3715 | 1000 bp upstream (*Spe*I-*Swa*I) & downstream (*Pac*I-*Nsi*I) of *ppe36* cloned into pML2424 (This is the knockout plasmid for deletion of *Mtb* *ppe36*) | Hyg |
| pML3723 | 1000 bp upstream (*Spe*I-*Swa*I) & downstream (*Pac*I-*Nsi*I) of *rv3533 (ppe62)* cloned into pML2424 (This is the knockout plasmid for deletion of *Mtb* *ppe62*) | Hyg |
| pML3726 | 1000 bp upstream (*Spe*I-*Swa*I) & downstream (*Pac*I-*Nsi*I) of *rv0265* cloned into pML2424 (This is the knockout plasmid for deletion of *Mtb* *rv0265*) | Hyg |
| ***Expression Vectors*** | |  |
| pML3716 | *ppe36* cloned into PacI-HindIII digested pMN016 (pMN016 contains strong **psmyc** mycobacterial promoter, can replicate in both *E. coli* and *Mycobacterium*) | Hyg |
| pML3724 | *ppe62* cloned into PacI-HindIII digested pMN016 (pMN016 contains strong **psmyc** mycobacterial promoter, can replicate in both *E. coli* and *Mycobacterium*) | Hyg |
| pML3727 | *rv0265* cloned into PacI-HindIII digested pMN016 (pMN016 contains strong **psmyc** mycobacterial promoter, can replicate in both *E. coli* and *Mycobacterium*) | Hyg |
| pML3731 | *pe22*-*ppe36* cloned into SphI-EcoRV digested pML1391 (C-term HA-His tagged PPE36 for subcellular localization) | Hyg |
| pML3732 | *ppe62* cloned into SphI-EcoRV digested pM1391 (C-term HA-His tagged PPE62 for subcellular localization) | Hyg |
| pML3733 | *rv0265* cloned into SphI-EcoRV digested pM1391 (C-term HA-His tagged Rv0265 for subcellular localization) | Hyg |
| pML1828 | *mbtG* cloned into SphI-EcoRV digested pMN016 (C-term HA-His tagged Rv0265 for subcellular localization) | Hyg |
| pML2109 | *rv0888* cloned into PacI-ScaI digested pMN016 (C-term HA-His tagged Rv0265 for subcellular localization) | Hyg |

**Table S1. Plasmids**

| **Name** | **Description** | **Abx Marker** |
| --- | --- | --- |
| ***Protein Purification Vectors*** | |  |
| pML3720 | *pe22-ppe36* cloned into XbaI-XhoI digested pET21a+ (6His-tagged PPE36 for protein purification) | Amp |
| pML3729 | *rv0265* without TAT signal (-24 bp) cloned into NdeI-HindIII digested pET21a+ (6His-tagged Rv0265 for protein purification) | Amp |
| pML3730 | *ppe62* cloned into NdeI-HindIII digested pET21a+ (6His-tagged PPE62 for protein purification) | Amp |
| pML3754 | *ideR* cloned into *NdeI*-*HindIII* digested pET21a+ (6His-tagged IdeR for protein purification) | Amp |
| pML3755 | *mhuD* cloned into *NdeI*-*HindIII* digested pET21a+ (6His-tagged MhuD for protein purification) | Amp |

**Table S1. Plasmids continued**
