# Supplementary material for: PPE Surface Proteins Are Required for Heme Utilization by Mycobacterium tuberculosis
Source: mBio. 2017 Jan 24;8(1):e01720-16. doi: 10.1128/mBio.01720-16 (PMC5263243; doi:10.1128/mBio.01720-16)
Supplement: TABLE S2 [file mbo001173164st9.docx]

| **Name** | **Sequence** | **Description** |
| --- | --- | --- |
| **Gene Deletion & Validation Primers** |  |  |
| ppe36-UF/SpeI | atat**ACTAGT**ACCAGCCGCCGGCTGAGGTC | Forward primer for amplifying upstream region of *ppe36*, contains SpeI restriction site |
| ppe36-UR/SwaI | atat**ATTTAAAT**AATTCGTTACTCCCTTGTAC | Reverse primer for amplifying upstream region of *ppe36*, contains SwaI restriction site |
| ppe36-DF/PacI | atat**TTAATTAA**TTCGGGAACATCCTAAGAAA | Forward primer for amplifying downstream region of *ppe36*, contains PacI restriction site |
| ppe36-DR/NsiI | atat**ATGCAT**TCGGCAGTTGGCCAATGTCT | Reverse primer for amplifying downstream region of *ppe36*, contains NsiI restriction site |
| ppe62-UF/SpeI | atat**ACTAGT**GCTGATAGCGACCAACTTTT | Forward primer for amplifying upstream region of *ppe62*, contains SpeI restriction site |
| ppe62-UR/SwaI | atat**ATTTAAAT**TTTCGCTTTGGTGCGGTATC | Reverse primer for amplifying upstream region of *ppe62*, contains SwaI restriction site |
| ppe62-DF/PacI | atat**TTAATTAA**GACCCCGTCCCTTCCCGACC | Forward primer for amplifying downstream region of *ppe62*, contains PacI restriction site |
| ppe62-DR/NsiI | atat**ATGCAT**TGACCCACGGCGACGGCCTG | Reverse primer for amplifying downstream region of *ppe62*, contains NsiI restriction site |
| rv0265-UF/SpeI | atat**ACTAGT**CGTTCACGGTCGGATCGGTG | Forward primer for amplifying upstream region of *rv0265*, contains SpeI restriction site |
| rv0265-UR/SwaI | atat**ATTTAAAT**GCATTTGCTAAGGCACCTCT | Reverse primer for amplifying upstream region of *rv0265*, contains SwaI restriction site |
| rv0265-DF/PacI | atat**TTAATTAA**cggcggctgcatccctgtcg | Forward primer for amplifying downstream region of *rv0265*, contains PacI restriction site |
| rv0265-DR/NsiI | atat**ATGCAT**tcggaaaaatgatcgaccac | Reverse primer for amplifying downstream region of *rv0265*, contains NsiI restriction site |
| ppe36-VF | CTACCAGCGAGTACAAGGGA | Forward primer for validation of deletion of *ppe36* |
| ppe36-VR | GCCCCCCGTTTCTTAGGATG | Reverse primer for validation of deletion of *ppe36* |
| ppe62-VF | TATGGCATTCGATACCGCAC | Forward primer for validation of deletion of *ppe62* |
| ppe62-VR | ACTGATTGTCAGGTCGGGAA | Reverse primer for validation of deletion of *ppe62* |
| rv0265-VF | CGAGACTGCCAGAGGTGCCT | Forward primer for validation of deletion of *rv0265* |
| rv0265-VR | CGGTAACGCGCGCGATACCC | Reverse primer for validation of deletion of *rv0265* |

**Table S2. Primers**

| **Name** | **Sequence** | **Description** |
| --- | --- | --- |
| **Complementation & Expression Primers** |  |  |
| ppe36-016Clone/F | atat**TTAATTAA**GGGAGAACAATGCCCAATTTCTGGGCGTT | Forward primer contains PacI restriction site, with optimized RBS sequence from pMN016, 20 nt hybridizing to ppe36, to clone ppe36 into pMN016 |
| ppe36-016Clone/R | atat**AAGCTT**TCAAAACTTAGGATGTTCCT | Reverse primer contains HindIII restriction site, 20 nt hybridinzing to ppe36, to clone ppe36 into pMN016 |
| ppe62-016Clone/F | atat**TTAATTAA**GGGAGAACAATGAACTATGCGGTATTGCC | Forward primer contains PacI restriction site, with optimized RBS sequence from pMN016, 20 nt hybridizing to ppe62, to clone ppe62 into pMN016 |
| ppe62-016Clone/R | atat**AAGCTT**TCAGTTCCCGAACCCCGACC | Reverse primer contains HindIII restriction site, 20 nt hybridinzing to ppe62, to clone ppe62 into pMN016 |
| rv0265-016Clone/F | atat**TTAATTAA**GGGAGAACAGTGCGACAGGGATGCAGCCG | Forward primer contains PacI restriction site, with optimized RBS sequence from pMN016, 20 nt hybridizing to rv0265, to clone rv0265 into pMN016 |
| rv0265-016Clone/R | atat**AAGCTT**TCATGCGCCCAAGATCTGGC | Reverse primer contains HindIII restriction site, 20 nt hybridinzing to rv0265, to clone rv0265 into pMN016 |
| ppe36-HA/F | atatGCATGCTCTTTGAGTTTCGAGGAGGAG | Forward primer contains SphI restriction site, 20 nt hybridizing to pe22, to clone pe22-ppe36 into pML1391 |
| ppe36-HA/R | atatGATATCAAACTTAGGATGTTCCTTGT | Reverse primer contains EcoRV restriction site, no stop codon, 20 nt hybridinzing to ppe36, to clone pe22-ppe36 into pML1391 |
| ppe62-HA/F | atatGCATGCGGGAGAACAATGAACTATGCGGTATTGCC | Forward primer contains SphI restriction site, with optimized RBS sequence from pMN016, 20 nt hybridizing to ppe62, to clone ppe62 into pML1391 |
| ppe62-HA/R | atatGATATCGTTCCCGAACCCCGACCGCT | Reverse primer contains EcoRV restriction site, no stop codon, 20 nt hybridinzing to ppe62, to clone ppe62 into pML1391 |
| rv0265-HA/F | atatGCATGCGGGAGAACAGTGCGACAGGGATGCAGCCG | Forward primer contains SphI restriction site, with optimized RBS sequence from pMN016, 20 nt hybridizing to rv0265, to clone rv0265 into pML1391 |
| rv0265-HA/R | atatGATATCTGCGCCCAAGATCTGGCTGA | Reverse primer contains EcoRV restriction site, no stop codon, 20 nt hybridinzing to rv0265, to clone rv0265 into pML1391 |

**Table S2. Primers continued**

| **Name** | **Sequence** | **Description** |
| --- | --- | --- |
| **Protein Purification Primers** |  |  |
| pe-ppe21aClone/F | atat**CATATG**TCTTTGAGTTTCGAGGAGGAG | Forward primer contains NdeI restriction site, replacing nativewith 20 nt hybridizing to pe22, to clone pe22-ppe36 into pET21a+ |
| pe-ppe21aClone/R | atat**AAGCTT**AAACTTAGGATGTTCCTTGT | Reverse primer contains HindIII restriction site, 20 nt hybridinzing to ppe36, replacing native stop codon, to clone pe22-ppe36 into pET21a+ |
| rv0265-21aClone/F | atat**CATATG**CGACAGGGATGCAGCCGCCG | Forward primer contains NdeI restriction site, replacing first 24 nt up to TAT signal, with 20 nt hybridizing to rv0265, to clone rv0265 into pET21a+ |
| rv0265-21aClone/R | atat**AAGCTT**TGCGCCCAAGATCTGGCTGA | Reverse primer contains HindIII restriction site, 20 nt hybridinzing to rv0265, replacing native stop codon, to clone rv0265 into pET21a+ or pML1970 |
| ppe62-21aClone/F | atat**CATATG**AACTATGCGGTATTGCCGCC | Forward primer contains NdeI restriction site, replacing native start codon, with 20 nt hybridizing to ppe62, to clone ppe62 into pET21a+ |
| ppe62-21aClone/R | atat**AAGCTT**GTTCCCGAACCCCGACCGCT | Reverse primer contains HindIII restriction site, 20 nt hybridinzing to ppe62, replacing native stop codon, to clone ppe62 into pET21a+ or pML1970 |
| ideR-21aClone/F | atat**CATATGCACCACCACCACCACCAC**AACGAGTTGGTTGATACCACCGAG | Forward primer contains NdeI restriction site, 6His, replacing native start codon, with 24 nt hybridizing to ideR, to clone ideR into pET21a+ |
| ideR-21aClone/R | atat**AAGCTT**TCAGACTTTCTCGACCTTGA | Reverse primer contains HindIII restriction site, 20 nt hybridinzing to ideR, to clone ideR into pET21a+ |
| mhuD-21aClone/F | atat**CATATGCACCACCACCACCACCAC**CCAGTGGTGAAGATCAACGCAATC | Forward primer contains NdeI restriction site, 6His, replacing native start codon, with 24 nt hybridizing to mhuD, to clone mhuD into pET21a+ |
| mhuD-21aClone/R | atat**AAGCTT**TTATGCAGTCTTGCCGGTCC | Reverse primer contains HindIII restriction site, 20 nt hybridinzing to mhuD, to clone mhuD into pET21a+ |

**Table S2. Primers continued**
