# Supplementary material for: PPE Surface Proteins Are Required for Heme Utilization by Mycobacterium tuberculosis
Source: mBio. 2017 Jan 24;8(1):e01720-16. doi: 10.1128/mBio.01720-16 (PMC5263243; doi:10.1128/mBio.01720-16)
Supplement: TABLE S3 [file mbo001173164st10.docx]

| **Strain** | **Parent strain and relevant genotype** | **Source or reference** |
| --- | --- | --- |
| *E. coli* DH5α | *recA1; endA1; gyrA96; thi; relA1; hsdR17(rK-;mK+); supE44; φ80*Δ*lacZ*Δ*M15;* Δ*lacZYA-argF; UE169* | (3) |
| *M. tuberculosis* mc^2^6206 | H37Rv derivative; Δ*leuCD* Δ*panCD*; avirulent *M. tuberculosis* | Dr. Jacobs (4) |
| H37Rv | wild-type *Mycobacterium tuberculosis* | ATCC# 27294 |
| H37Rv ML2411 | H37Rv derivative; Δ*ppe36*::*loxP* | This study |
| H37Rv ML2414 | H37Rv ML2411 derivative; Δ*ppe36*::*loxP*; pML3716; hyg^R^ | This study |
| *M. tuberculosis* ML2408 | mc^2^6206 derivative; pML3731; hyg^R^ | This study |
| *M. tuberculosis* ML2409 | mc^2^6206 derivative; pML3732; hyg^R^ | This study |
| *M. tuberculosis* ML2410 | mc^2^6206 derivative; pML3733; hyg^R^ | This study |
| *M. tuberculosis* ML2411 | mc^2^6206 derivative; Δ*ppe36*::*loxP* | This study |
| *M. tuberculosis* ML2412 | mc^2^6206 derivative; Δ*ppe62*::*loxP* | This study |
| *M. tuberculosis* ML2413 | mc^2^6206 derivative; Δ*rv0265*::*loxP* | This study |
| *M. tuberculosis* ML2414 | ML2411 derivative; Δ*ppe36*::*loxP*; pML3716; hyg^R^ | This study |
| *M. tuberculosis* ML2415 | ML2412 derivative; Δ*ppe62*::*loxP*; pML3724; hyg^R^ | This study |
| *M. tuberculosis* ML2416 | ML2413 derivative; Δ*rv0265*::*loxP*; pML3727; hyg^R^ | This study |
| *M. tuberculosis* ML2425 (Mtb Tn1) | mc^2^6206 derivative; *rv0265c-rv0266c*::*tn*; transposon insertion in intergenic region of *rv0265c-rv0266c* in nts 317514 and 317515; kan^R^ | This study |
| *M. tuberculosis* ML2426 (Mtb Tn2) | mc^2^6206 derivative; *rv2107-rv2108*::*tn*; transposon insertion in intergenic region of *rv2107* (*pe22*) and *rv2108* (*ppe36*) in nts 2367708 and 2367709; kan^R^ | This study |
| *M. tuberculosis* ML2427 (Mtb Tn3) | mc^2^6206 derivative; *rv0161*::*tn*; transposon insertion in nts 190701 and 190702; kan^R^ | This study |
| *M. tuberculosis* ML2428 (Mtb Tn4) | mc^2^6206 derivative; *rv0197*::*tn*; transposon insertion in nts 233336 and 233337; kan^R^ | This study |
| *M. tuberculosis* ML2429 (Mtb Tn5) | mc^2^6206 derivative; *rv3533*::*tn*; transposon insertion in start codon of *rv3533* (*ppe62*) in nts 3972452 and 3972454; kan^R^ | This study |

**Table S3. Strains.**

| **Strain** | **Parent strain and relevant genotype** | **Source or reference** |
| --- | --- | --- |
| *M. tuberculosis* ML2430 (Mtb Tn6) | mc^2^6206 derivative; *rv2385*::*tn*; transposon insertion in nts 2678016 and 2678017; kan^R^ | This study |
| *M. tuberculosis* ML2431 (Mtb Tn7) | mc^2^6206 derivative; *rv2950*::*tn*; transposon insertion in nts 3301359 and 3302455; kan^R^ | This study |
| *M. tuberculosis* ML2432 (Mtb Tn8) | mc^2^6206 derivative; *rv2947*::*tn*; transposon insertion in nts 3296444 and 3296445; kan^R^ | This study |
| *M. tuberculosis* ML2433 (Mtb Tn9) | mc^2^6206 derivative; *rv1752*::*tn*; transposon insertion in nts 1981157 and 1981158; kan^R^ | This study |
| *M. tuberculosis* ML2434 (Mtb Tn10) | mc^2^6206 derivative; *rv1131*::*tn*; transposon insertion in nts 1256313 and 1256314; kan^R^ | This study |
| *M. tuberculosis* ML2435 | mc^2^6206 derivative; pML1828; hyg^R^ | This study |
| *M. tuberculosis* ML2436 | mc^2^6206 derivative; pML2109; hyg^R^ | This study |
| *M. tuberculosis* ML2437 | mc^2^6206 derivative; pML1391; hyg^R^ | This study |
| *M. tuberculosis* ML2438 | ML2411 derivative; Δ*ppe36*::*loxP*; pML3724; hyg^R^ | This study |
| *M. tuberculosis* ML2439 | ML2411 derivative; Δ*ppe36*::*loxP*; pML3727; hyg^R^ | This study |
| *M. tuberculosis* ML2440 | ML2412 derivative; Δ*ppe62*::*loxP*; pML3716; hyg^R^ | This study |
| *M. tuberculosis* ML2441 | ML2412 derivative; Δ*ppe62*::*loxP*; pML3727; hyg^R^ | This study |
| *M. tuberculosis* ML2442 | ML2413 derivative; Δ*rv0265*::*loxP*; pML3716; hyg^R^ | This study |
| *M. tuberculosis* ML2443 | ML2413 derivative; Δ*rv0265*::*loxP*; pML3724; hyg^R^ | This study |
| *M. tuberculosis* ML2444 | ML2411 derivative; Δ*ppe36*::*loxP*; pML3731; hyg^R^ | This study |
| *M. tuberculosis* ML2445 | ML2412 derivative; Δ*ppe62*::*loxP*; pML3724; hyg^R^ | This study |
| *M. tuberculosis* ML2446 | ML2413 derivative; Δ*rv0265*::*loxP*; pML3732; hyg^R^ | This study |

**Table S3. Strains continued**
